# Exploring Visceral Fat as a Screening Marker for Cardiometabolic Risk in Children and Adolescents

**DOI:** 10.3390/children12030308

**Published:** 2025-02-28

**Authors:** Xia Wang, Hong Cheng, Jingfan Xiong, Junting Liu, Hongbo Dong, Liwan Fu, Xiangjun Xie, Xinying Shan, Xiaoyuan Zhao, Yinkun Yan, Pei Xiao, Jie Mi

**Affiliations:** 1 Center for Non-Communicable Disease Management, Beijing Children’s Hospital, Capital Medical University, National Center for Children’s Health, Beijing 100035, China; xxlwangxia@mail.ccmu.edu.cn (X.W.); h018385@mail.ccmu.edu.cn (H.D.); liwanfu@mail.ccmu.edu.cn (L.F.); 112023010335@mail.ccmu.edu.cn (X.X.); ykyan2024@mail.ccmu.edu.cn (Y.Y.); 2 Department of Epidemiology, Capital Institute of Pediatrics, Beijing 100035, China; lxbxyjs@shouer.com.cn (H.C.); junting_liu@shouer.com.cn (J.L.); monicashan_2007@163.com (X.S.); xyzhao2009@163.com (X.Z.); 3 Department of Child and Adolescent Chronic Disease Prevention and Control, Shenzhen Center for Chronic Disease Control, Shenzhen 518000, China; smbyy@wjw.sz.gov.cn

**Keywords:** cardiometabolic risk, visceral fat area, cutoffs, obesity, children, adolescents

## Abstract

**Objective:** To establish and validate age- and sex-specific visceral fat area (VFA) cutoff values for the effective identification of cardiometabolic risk (CMR) in children and adolescents. **Methods:** A cross-sectional study involving 8133 participants was conducted to derive age- and sex-specific VFA cutoffs, which were validated in a longitudinal cohort comprising 10,805 individuals. The predictive performance of the derived VFA cutoffs for CMR was evaluated using the area under the receiver operating characteristic curve (AUC). Additionally, logistic regression models were utilized to calculate the relative risk (RR) of CMR associated with elevated VFA levels. **Results:** The 75th percentile of the VFA was identified as the optimal cutoff for screening for hypertension, hyperglycemia, dyslipidemia, and CMR clustering in boys. In girls, the 75th percentile was optimal for screening hypertension, dyslipidemia, and CMR clustering, while the 80th percentile proved best for hyperglycemia. No significant difference in predicative performance was observed between the optimal and simplified VFA cutoffs. Longitudinal validation demonstrated that individuals exceeding the VFA cutoff had a significantly higher risk for CMR, with RRs ranging from 1.33 to 3.89 (all *p* < 0.001) for boys and from 1.63 to 3.16 (all *p* < 0.001) for girls. Notably, normal-weight boys with VFA above the cutoff had a significantly higher CMR risk compared to their peers in other weight status categories. **Conclusions:** Both the optimal and simplified VFA cutoffs are robust tools for screening CMR in Chinese children and adolescents, with significant implications for early intervention strategies.

## 1. Introduction

Obesity or excess fat has been verified as an increased risk of cardiometabolic diseases [1]. Cardiovascular events predominantly occur in adults, the pathological changes associated with these events often originate from cardiovascular and metabolic abnormalities in early childhood [2,3]. The International Childhood Cardiovascular Cohort (i3C) Consortium has confirmed that childhood risk factors are associated with cardiovascular events in adulthood [4,5]. Components of metabolic risk factors, including high total cholesterol, low high-density lipoprotein, and elevated low-density lipoprotein, are frequently associated with being overweight or central obesity [6]. Consequently, early screening and intervention for obesity in children and adolescents may be crucial in delaying the development of cardiometabolic diseases.

The definition and diagnostic criteria of clinical obesity highlight that body mass index (BMI) does not distinguish between fat and lean mass or account for variations in body fat distribution [7]. BMI-based measures can both underestimate and overestimate adiposity, offering limited information about overall health [8]. Ectopic fat accumulation, primarily in visceral fat, is a major contributor to obesity-related health complications. This fat secretes various bioactive molecules, including inflammatory cytokines, free fatty acids, and adipokines, which can impair insulin sensitivity, promote endothelial dysfunction, and elevate blood pressure [9,10]. Empirical evidence indicates that visceral fat accumulation is a stronger and more consistent predictor of cardiometabolic risk than overall body fat. Visceral fat area (VFA) provides an accurate measure of abdominal adiposity and warrants widespread adoption [11]. The efficacy of dual-energy X-ray absorptiometry (DXA) in assessing visceral fat is comparable to established standard techniques like CT and MRI, and DXA has been validated in both adults and children due to its low radiation exposure [12,13]. Currently, there is a lack of established risk cutoff points for cardiovascular metabolic risk (CMR) screening using DXA-based VFA in children and adolescents.

This study aims to determine the optimal VFA cutoff for identifying CMR in children and adolescents aged 6–18 years, offering a reference for early detection and prevention of cardiovascular metabolic diseases during this critical developmental period.

## 2. Method

### 2.1. Study Population

Data for determining VFA risk cutoffs were derived from the China Child and Adolescent Cardiovascular Health (CCACH) Study, a large-scale population-based cross-sectional study conducted between 2013 and 2015. The study aimed to recruit a nationally representative sample of children and adolescents aged 6–18 years from urban areas across China. Briefly, China was stratified into northern and southern regions based on the Qinling–Huaihe line, considering variations in climate, economic development, and lifestyle habits. Four cities were selected from the northern region (Beijing, Changchun, Jinan, and Yinchuan) and two from the southern region (Shanghai and Chongqing). Schools within each city were randomly selected to ensure representation across sex, age, and socioeconomic status [14,15]. The inclusion criteria comprised children and adolescents aged 3 to 17 years who had not undergone major surgery in the past year, had no physical disabilities or deformities, and had not used hormonal medications for three consecutive months. All students from the selected schools were invited to participate in a clinical examination, including a questionnaire survey, anthropometric measurements, blood sample collection, and DXA scan.

The validation portion used data from the School Cardiovascular and Bone Health Program (SCVBH), a prospective study aimed at investigating the risk factors associated with cardiovascular and bone health in school-aged children. The inclusion criteria comprised children and adolescents who had not undergone major surgery in the past year, had no physical disabilities or deformities, and had not used hormonal medications for three consecutive months. Its protocol and baseline characteristics of the study population have been reported elsewhere [16]. A stratified cluster sampling design was implemented across 30 schools (8 primary, 21 secondary, and 1 combined K–12 institution) in Beijing’s Dongcheng, Tongzhou, Fangshan, and Miyun districts. Between November 2017 and September 2018, all students in grades 1–4 of primary school, first-year junior high, and first-year senior high were eligible for baseline assessment. Participants completed self-administered questionnaires, physical examinations, and biospecimen collection. Follow-up evaluations were conducted through December 2019, with 12,984 individuals completing identical assessments. The protocol encompassed demographic data collection, anthropometric measurements, and body composition analysis.

**Figure 1:**
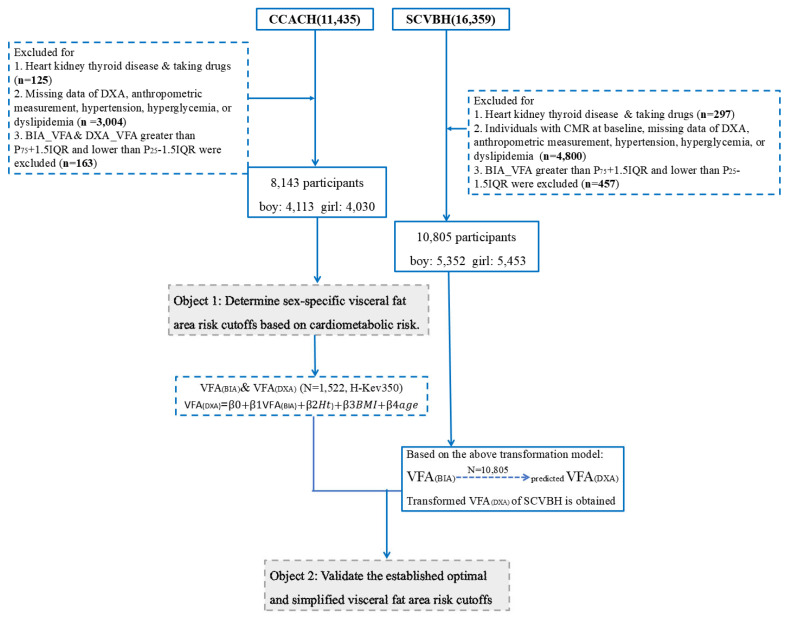
Flowchart of inclusion and exclusion for CCACH, the China Child and Adolescent Cardiovascular Health; SCVBH, the School-based Cardiovascular and Bone Health Promotion Program; DXA, dual-energy X-ray absorptiometry; VFA, visceral fat area.

This study initially selected 8133 participants from the CCACH and 10,805 participants from the SCVBH and applied the following exclusion criteria at baseline: (1) had heart, kidney, or thyroid disease or were taking hormone medication; (2) lacked data for anthropometry and CMR; and (3) had extreme VFA values (P_75_ + 1.5IQR ≥ and ≤P_25_ − 1.5IQR) (details shown in Figure 1). The work has been reported in line with the STROCSS criteria [17].

### 2.2. Anthropometric and Body Composition Measurements

Body weight and height were measured twice using a calibrated digital scale (Jianmin II, China Institute of Sport Science, Beijing, China) with participants wearing light clothing and no footwear. Measurements were recorded to the nearest 0.1 kg and 0.1 cm, respectively. Body mass index (BMI) was calculated as weight (kg) divided by height squared (m^2^). Weight status was categorized into normal weight, overweight, and obesity based on age- and sex-specific BMI cutoffs established by the International Obesity Task Force (IOTF) [18,19,20]. Blood pressure (BP) was measured three times at 1–2 min intervals on the right arm using an Omron HEM-7012 electronic sphygmomanometer (Omron Co., Kyoto, Japan), with cuff sizes selected based on arm circumference (small, medium, or large for 13–22 cm, 22–32 cm, and 32–42 cm, respectively). The mean value was used for analysis. Hypertension was defined as systolic and/or diastolic BP exceeding the 95th percentile for sex, age, and height, or the use of antihypertensive medication [21].

Dual-energy X-ray absorptiometry (DXA) scans were performed using Hologic Discovery fan-beam densitometers (Models A, W, and Wi, Marlborough, MA, USA) following standardized protocols in the CCACH study. Rigorous quality control measures were implemented, including the use of consistent DXA machines and trained technicians across all centers, as well as daily phantom calibrations prior to participant scans. Scans were analyzed using APEX software (version 3.0), which automatically quantified regional fat mass. VFA was assessed by scanning the android region, defined as the area between the lumbar spine midpoint and the pelvic crest.

Bioelectrical impedance analysis (BIA) (H-Kev350, Sihaihuachen, Beijing, China) uses 8-point contact electrodes to perform electrical impedance measurements at 5 body segments using 3 different frequencies (5, 50, and 500 kHz) in SCVBH. Subjects were required to fast or fast for 2 h before the test. Large amounts of water and strenuous activities were prohibited. The subjects stepped on the test table with their bare feet and pointed their heel on the foot electrode. At the same time, the electrode was shaken with both hands, and their arms were opened at approximately 30°.

### 2.3. Laboratory Analysis

Venous blood samples were collected, after a 12 h overnight fast, into vacuum tubes containing ethylenediaminetetraacetic acid (EDTA). Plasma and serum were separated within 30 min and stored at −80 °C. Samples were then shipped on dry ice to the central clinical laboratory of the Capital Institute of Pediatrics in Beijing. Fasting blood glucose (FBG), HDL-C, TG, and uric acid (UA) were measured using a Hitachi 7080 biochemistry autoanalyzer (Hitachi, Tokyo, Japan). Total cholesterol (TC) and TG were measured using the enzymatic method (Sekisui Medical, Tokyo, Japan), while HDL-C and LDL-C were analyzed by the direct method (Sekisui Medical, Tokyo, Japan). Fasting glucose was measured using the hexokinase method (Biosino Biotechnology, Beijing, China). Hyperglycemia was defined as a fasting plasma glucose ≥5.6 mmol/L or the use of antidiabetic medications [22]. Individuals were classified as having dyslipidemia if their lipid levels (TC, TG, LDL-C, or HDL-C) exceeded the sex- and age-specific cutoffs for Chinese children and adolescents [23]. CMR clustering was defined as the presence of two or more risk factors for hypertension, hyperglycemia, or dyslipidemia.

### 2.4. Statistical Analysis

Categorical data were summarized as proportions with 95% confidence intervals (CIs), while continuous data were expressed as means ± standard deviations (SDs) or medians with interquartile ranges (IQRs). Group differences in categorical and continuous variables were assessed using the χ^2^ test and *t* test, respectively. The accuracy of VFA in identifying cardiometabolic risk (CMR) and CMR clustering was evaluated using receiver operating characteristic (ROC) analysis. Sex-specific VFA percentiles and z-scores were constructed by age using the lambda-mu-sigma (LMS) method [24,25]. The z-scores were calculated using the following formula:

$$Z=\frac{{\left(\frac{X}{M}\right)}^{L}-1}{L\times S}\left(if\neq 0\right)\;or\;Z=\frac{in\left(\frac{X}{M}\right)}{S}\left(if=0\right)$$

Percentiles were obtained from z-scores, and the 75th to 95th percentiles correspond to z-scores of 0.673 to 1.635, respectively. The present study employed age- and sex-specific z-scores of DXA-VFA to predict hypertension, hyperglycemia, dyslipidemia, and CMR clustering. The predictive ability was quantified using the area under the ROC curve (AUC), with ROC curves generated from the same set of subjects for all VFA measurements. AUCs for the optimal and simplified cutoffs were compared using the DeLong test. To streamline VFA thresholds, optimal cutoffs were consolidated into four age groups (6–8 years, 9–11 years, 12–15 years, and 16–18 years), calculated as the means of the respective cutoffs within each group. Logistic regression analyses assessed the relative risk associated with simplified and optimal cutoffs for identifying CMR and its clustering.

Scatter plots evaluated sex-specific trends in DXA-VFA and BIA-VFA, as shown in Supplementary Figure S1. Linear regression analysis quantified the relationship between BIA-measured VFA and anthropometric factors (age, height, and BMI) obtained via DXA. Bland–-Altman plots assessed the agreement between DXA-VFA and predicted values, with differences plotted against their mean values for each body composition measurement. Limits of agreement (LOA) were calculated as the mean difference (bias) ± 1.96 SD (95% confidence interval) to test the agreement between DXA-measured and BIA-predicted VFA values. The conversion of BIA-VFA to DXA-VFA in the SCVBH was derived using these calculations, with specific equations used for boys and girls.

DXA-VFA-_predicted boy_ = 3.885 + 0.295 × BIA-VFA−1.036 age + 1.557 × BMI + 0.066 × height

DXA-VFA-_predicted girl_ = −17.612 + 0.287 × BIA-VFA−1.109 × age + 1.960 × BMI + 0.062 × height

All the statistical analyses were conducted using R software, version 4.3.0 (http://www.R-project.org, accessed on 3 February 2024). A 2-tailed *p*-value < 0.05 was considered to indicate statistical significance.

## 3. Results

Table 1 presents data from 8133 participants (mean age = 11.8 years, SD = 3.3 years) in the CCACH study and 10,805 participants (mean age = 11.0 years, SD = 3.3 years) in the SCVBH study, assessed using BIA. In the CCACH study, the prevalence of conditions was as follows: hypertension 18.3%, hyperglycemia 27.3%, dyslipidemia 13.9%, and two or more CMRs 11.2%. In the SCVBH study, the prevalence of conditions was as follows: hypertension 23.1%, hyperglycemia 10.9%, dyslipidemia 13.3%, and two or more CMRs 9.0%. Boys exhibited higher prevalence of CMRs and clustering than girls in both studies (all *p* < 0.001). Additionally, 1522 participants in the CCACH study were evaluated for VFA using DXA and BIA, with their characteristics detailed in Supplementary Table S1.

### 3.1. DXA-Based Optimal Risk and Simplified Cutoffs of VFA for Identifying CMR and Its Clustering

Table 2 showed the area under the ROC curves and the test characteristics of the DXA-based optimal VFA risk cutoffs for identifying CMR and its clustering. The 75th percentile of VFA is the most effective threshold for screening hypertension, hyperglycemia, dyslipidemia, and CMR clustering in boys. For girls, the 75th percentile is used for hypertension, dyslipidemia, and CMR clustering, while the 80th percentile is applied for hyperglycemia. Corresponding cutoff values are presented in Table 3. The optimal cutoffs were further simplified by age group based on the average values of sex- and age-specific VFA cutoffs, with detailed thresholds provided in Supplementary Table S2. The subgroup analysis of VFA risk cutoffs revealed that the Han ethnicity had higher values than others. There were no statistically significant differences across various dietary, exercise, and family economic status subgroup (Supplementary Figure S1).

### 3.2. The Performance of the Transformed VFA for Identifying the CMR and Its Cluster

The consistency of the DXA-VFA and the predicted VFA is presented in Bland-Altman plots (Supplementary Figure S2). The predicted VFAs were used to validate the performance of the optimal and simplified cutoffs for predicting the occurrence of CMR and its cluster. Table 4 presents the AUCs for identifying hypertension, hyperglycemia, dyslipidemia, and CMR ≥ 2. The optimal and simplified VFA cutoffs in both boys and girls ranged from 0.619 (95% CI: 0.590–0.638) to 0.653 (95% CI: 0.633–0.673). The screening performance of optimal and simplified VFA did not have any statistical differences (*p* > 0.05), except for hypertension among boys (*p* = 0.008).

### 3.3. The Validation of Optimal and Simplified VFA-DXA Cutoffs in the SCVBH

**Figure 2:**
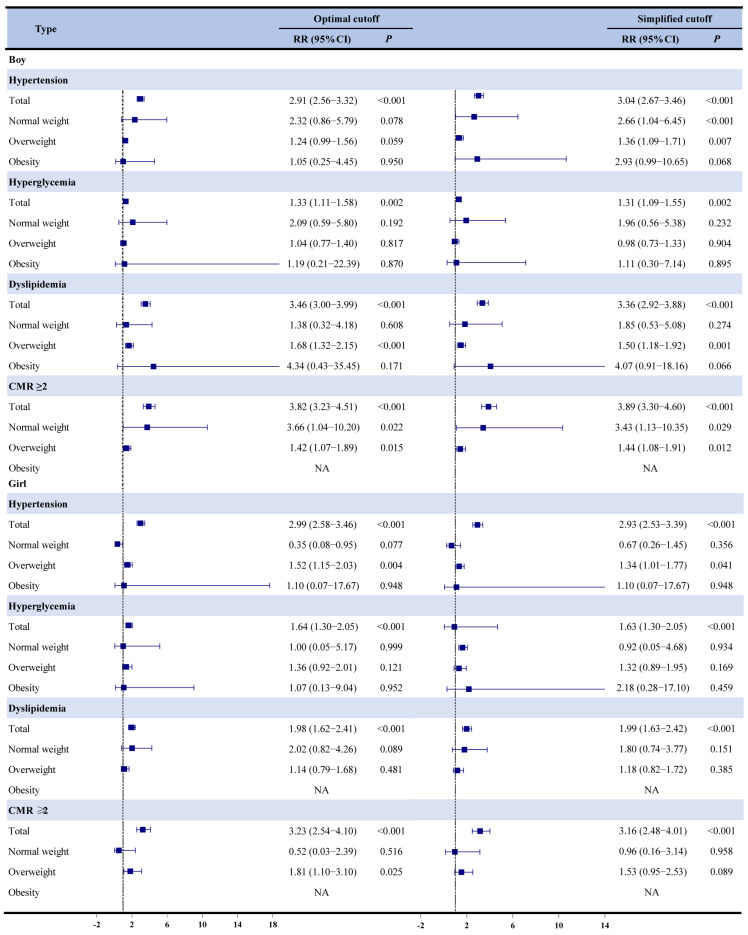
The association of optimal and simplified cutoffs to identify CMR and its clustering after 2 years of follow-up in SCVBH RR, relative risk.

Figure 2 presented the estimated relative risks (RRs) for hypertension in boys: 2.91 (95% CI: 2.56–3.32, *p* < 0.001) for the optimal cutoff and 3.03 (95% CI: 2.67–3.36, *p* < 0.001) for the simplified cutoff. For hyperglycemia, the RRs were 1.33 (95% CI: 1.11–1.58, *p* = 0.002) and 1.31 (95% CI: 1.09–1.55, *p* = 0.002). Dyslipidemia showed an RR of 3.36 (95% CI: 3.00–3.99, *p* < 0.001) for both cutoffs, for CMR ≥ 2, the RRs were 3.82 (95% CI: 3.23–3.51, *p* < 0.001) and 3.89 (95% CI: 3.30–3.60, *p* < 0.001). In girls, similar findings demonstrated that DXA-VFA levels exceeding the established cutoffs were significantly associated with an increased risk of CMR and its clustering (all *p* < 0.001). Further verification showed that normal-weight boys exceeding the optimal and simplified cutoffs had a 3.66-fold greater risk (95% CI: 1.03–10.20) of hypertension, hyperglycemia, and CMR ≥ 2 compared to other subgroups.

## 4. Discussion

In this study, we determined DXA-based, age- and sex-specific VFA risk cutoffs and simplified thresholds for detecting CMR and its clustering. The 75th percentile in boys and girls was used to screen for hypertension, hyperglycemia, and CMR ≥ 2, while the 80th percentile in girls was used for hyperglycemia screening. The simplified thresholds were categorized into age groups: 6–8, 9–11, 12–15, and 16–18 for phenotyping. The screening performance of these cutoffs was assessed in a two-year longitudinal cohort.

In recent years, CMR in children and adolescents has been reported in various regions [2,26,27]. One particular concern is the decreasing age at the onset of diabetes in young people [28]. Anthropometric measures are indirect indicators of visceral fat, but their sensitivity for evaluating fat distribution may be insufficient [29]. Visceral fat has been demonstrated to be a direct and independent risk factor for the occurrence of cardiovascular diseases [30]. A study in the Czech Republic, based on a population aged 25–64, established effective VFA cutoffs through bioelectrical impedance measurement to assess visceral fat and cardiovascular metabolic risk [31]. Another study in South Korea, involving individuals aged 17–69, evaluated race-specific cutoff values for visceral fat area determined by computed tomography in relation to cardiovascular metabolic risk, with better validation compared to indicators such as BMI and waist circumference [32]. Currently, there is a lack of established screening cutoffs for directly assessing visceral fat based on DXA measurements in children and adolescents. VFA cutoffs contribute to effective clinical assessment of cardiovascular disease risk in the era of precision medicine.

Using CMR and its clustering as endpoints, the optimal age- and sex-specific screening cutoffs vary significantly across different BMI-based weight statuses. Screening results show that boys surpassing these thresholds have a higher risk of hypertension, hyperglycemia, hyperlipidemia, and CMR ≥ 2 than girls, regardless of gender or weight status. Consistent with our study, other research has shown that visceral fat is a strong predictor of cardiometabolic risk in prepubescent boys, while it performs poorly in girls [29,33]. It is well established that there are gender differences in using visceral fat to distinguish cardiometabolic risk in adults [34,35], and these differences are evident early in life, becoming more pronounced during puberty [36]. Factors such as sexual maturation [37] and skeletal growth [38] may explain the gender disparities in fat storage. Sex hormones and gender-specific molecular mechanisms influence glucose and lipid metabolism, which in turn affect cardiac energy metabolism and function, leading to varying levels of cardiovascular metabolic risk [39]. On this basis, we found that boys in the normal weight group exhibit a greater risk than those in other weight categories. Anika Nier et al. also reported that conservative metabolic abnormalities are associated with increased visceral fat in normal-weight children [40]. Therefore, in clinical practice, it is essential to enhance the screening of VFA risks in normal-weight boys. Additionally, when examining the differences in optimal cutoffs among subpopulations, the optimal VFA cutoffs in the Han population were found to be higher than those of other ethnic groups. However, since this study primarily included Han participants, the observed differences may be constrained by the small sample size of minority groups. Future research should explore these differences by matching various ethnic populations. It is important to suggest that the risk for minority groups exceeding these cutoffs may be greater. Other subgroup differences showed no statistically significant variations.

In addition to age- and sex-specific risk thresholds, we consolidated these percentiles into a simplified table. Compared to the optimal cutoff points, the simplified thresholds exhibited higher consistency and comparable performance in identifying CMR and its clustering, validating their utility for screening purposes. While the use of DXA in large epidemiological studies is often impractical, it remains relatively precise and applicable in clinical settings. This allows for the calibration of more convenient bioelectrical impedance analysis techniques based on these thresholds. Therefore, our simplified table facilitates an efficient and practical screening process for cardiovascular disease risk in children and adolescents in clinical practice.

Finally, the main strengths of our study were the large sample size, the derivation of simplified cutoffs, and the verification of the relative risk of CMR and its clustering in a cohort. There are also several limitations in terms of not being a good representation of the whole pediatric population of China because of the nonrandom sampling method. The association between visceral fat and adult cardiovascular endpoint events was not examined due to the short follow-up duration in the SCVBH cohort. Additionally, compared to MRI and CT measurements of visceral fat, DXA exhibits some degree of bias, particularly in overweight and obese children and adolescents; however, this bias is relatively minor [41,42].

In conclusion, the present study showed that the 75th percentile of VFA in boys and girls is the optimal cutoff for screening CMR and its clustering, but the 80th percentile is the optimal cutoff for hyperglycemia screening in girls. Additionally, simplified age-specific cutoff values for 6–8, 9–11, 12–15, and 16–18 years are proposed for practical use. Those may yield insight into the risks associated with child obesity and the initial phases of cardiovascular disease development, especially in normal-weight individuals.

## Figures and Tables

**Table 1:** Characteristics of study population.

Variables	CCACH				SCVBH			
	Boy (*n* = 4113)	Girl (*n* = 4030)	Overall (*n* = 8143)	*p*-Value	Boy (*n* = 5352)	Girl (*n* = 5453)	Overall (*n* = 10,805)	*p*-Value
Age, y	12.0 ± 3.7	12.4 ± 3.7	11.8 ± 3.4	<0.001	10.9 ± 3.4	11.0 ± 3.4	11.0 ± 3.4	0.245
BMI, kg/m^2^	19.9 ± 4.1	19.2 ± 3.5	19.5 ± 3.8	<0.001	20.2 ± 4.1	19.2 ± 3.8	19.7 ± 4.0	<0.001
Hight, cm	154.0 ± 18.8	150.0 ± 14.8	151.7 ± 17.1	<0.001	150.0 ± 19.3	136.0 ± 16.0	147.8 ± 17.8	<0.001
BIA_VFA	-	-	-	-	34.0 (21.5, 62.7)	44.6 (24.5, 62.7)	38.7 (22.9, 68.6)	<0.001
DXA_VFA *	43.9 (35.1, 55.9)	32.0 (20.7, 45.9)	38.9 (28.5, 51.9)	<0.001	42.2 (35.7, 55.8)	28.1 (19.6, 41.1)	37.0 (27.8, 49.2)	<0.001
Hypertension	809 (19.7)	683 (16.9)	1492 (18.3)	0.001	1503 (28.1)	1105 (20.3)	2608 (24.1)	<0.001
Hyperglycemia	1309 (32.4)	880 (22.2)	2189 (27.4)	<0.001	670 (13.6)	503 (13.4)	1173 (10.9)	<0.001
Dyslipidemia	804 (19.5)	413 (10.2)	1217 (14.9)	<0.001	1038 (19.4)	510 (9.4)	1548 (14.3)	<0.001
CMR ≥ 2	592 (14.7)	302 (7.6)	894 (11.2)	<0.001	676 (12.6)	298 (5.5)	974 (9.0)	<0.001

Abbreviations: BMI, body mass index; BIA-VFA, visceral fat area was measured by bioelectric impedance analysis; DXA-VFA, visceral fat area was measured by dual-energy X-ray absorptiometry; CMR, cardiometabolic risk. * DXA-VFA of SCVBH is the transformed data.

**Table 2:** Optimal cutoffs and test characteristics of VFA for screening CMR and its clustering.

Type	Boy				Girl			
	AUC	pct	Se	Sp	AUC	pct	Se	Sp
Hypertension	0.594	p75	40.0%	78.7%	0.561	p75	30.2%	82.1%
Hyperglycemia	0.522	p75	28.0%	76.4%	0.528	p80	29.4%	76.2%
Dyslipidemia	0.619	p75	44.2%	79.6%	0.571	p75	37.8%	76.4%
CMR ≥ 2	0.619	p75	45.3%	78.5%	0.600	p75	38.4%	81.6%

Abbreviations: AUC, area under the receiver operating characteristic curve; pct, percentile; Se, sensitivity; Sp, specificity; VFA, visceral fat area; CMR, cardiometabolic risk.

**Table 3:** The optimal VFA cutoffs for children and adolescents aged 6–18 years.

Age (Years)	VFA (75th)		VFA (80th)	
	Boys	Girls	Boys	Girls
6	44.96	29.39	48.97	34.27
7	46.77	30.69	51.25	35.89
8	49.82	33.20	54.79	38.72
9	54.08	36.38	59.46	42.17
10	57.54	39.57	63.18	45.52
11	58.65	42.58	64.39	48.61
12	58.06	45.11	63.74	51.13
13	57.00	47.23	62.55	53.13
14	56.35	49.35	61.72	55.10
15	56.76	51.65	61.92	57.29
16	58.26	53.55	63.23	59.15
17	59.07	54.25	63.89	59.85
18	57.94	53.94	62.63	59.58

Abbreviations: VFA, visceral fat area; 75th: the 75th percentile; 80th: the 80th percentile.

**Table 4:** The performance of optimal and simplified cutoffs to identify CMR and its clustering in SCVBH.

Type	Boy (AUC)		*p*-Value	Girl (AUC)		*p*-Value
	Optimal Cutoff	Simplified Cutoff		Optimal Cutoff	Simplified Cutoff	
Hypertension	0.610(0.596–0.624)	0.616(0.602–0.630)	0.008	0.529(0.510–0.547)	0.536(0.517–0.554)	0.187
Hyperglycemia	0.529(0.510–0.547)	0.527(0.509–0.546)	0.705	0.536(0.517–0.554)	0.535(0.517–0.554)	0.759
Dyslipidemia	0.634(0.618–0.651)	0.632(0.616–0.649)	0.503	0.565(0.544–0.587)	0.565(0.544–0.586)	0.916
CMR ≥ 2	0.650(0.630–0.670)	0.654(0.634–0.673)	0.239	0.623(0.594–0.652)	0.619(0.590–0.648)	0.354

Abbreviations: AUC, area under the receiver operating characteristic curve; CMR, cardiometabolic risk.

## Data Availability

Data can be obtained from the corresponding author (jiemi12@vip.sina.com).

## Supplementary Materials

The following supporting information can be downloaded at: https://www.mdpi.com/article/10.3390/children12030308/s1, Table S1: Characteristics of study population. Table S2: Simplified thresholds of VFA for boys and girls aged 6 to 18 years. Table S3: The association of simplified and optimal cutoffs to identify CMR and its clustering after 2 years of follow-up in SCVBH. Figure S1: The subgroup analysis of the DXA-based optimal VFA risk cutoffs. (A) The DXA-based optimal VFA risk cutoffs in different ethnic groups (*p* < 0.01). (B) The DXA-based optimal VFA risk cutoffs in different income (*p >* 0.05). (C) The DXA-based optimal VFA risk cutoffs in different diets (*p >* 0.05). (D) The DXA-based optimal VFA risk cutoffs in different physical activities (*p >* 0.05). Ethnic groups were classified into Han and minority populations (Han: 90.0% ). Family income was categorized based on annual income: greater than 100,000 yuan and less than or equal to 100,000 yuan(>100 million yuan: 24%). Dietary classifications were based on the consumption of five food categories over the past month: 1. Vegetables or fruits ≥ 1 time/day; 2. Meat and meat products (pork, beef, lamb, and poultry) ≥ 2 times/week; 3. Dairy products ≥ 1 time/day; 4. Soy products ≥ 1 time/day; 5. Sugary beverages < 1 time/week. Individuals meeting four or more of these criteria were classified as meeting dietary standards/balance of nutrition; those who did not were classified as not meeting standards/nutritional imbalance (Dietary standard: 67.5%). Physical activity was defined as sufficient if individuals engaged in an average of 60 minutes or more of moderate to vigorous exercise per day over the past month; otherwise, it was considered insufficient (>1 h/d: 23.6%). Abbreviations: VFA, visceral fat area. Figure S2: Scatter plot of sex-specific VFA trends between DXA and BIA measurements. Abbreviations: BIA-VFA, Visceral fat area was measured by bioelectric impedance analysis; DXA-VFA, Visceral fat area was measured by dual-energy X-ray absorptiometry. Figure S3: Bland–Altman analysis comparing the difference between VFA from DXA and predicted DXA (n = 1522). Abbreviations: DXA, dual-energy X-ray absorptiometry.
