# Supplementary material for: Exploring Visceral Fat as a Screening Marker for Cardiometabolic Risk in Children and Adolescents
Source: Children (Basel). 2025 Feb 28;12(3):308. doi: 10.3390/children12030308 (PMC11941014; doi:10.3390/children12030308)
Supplement: Supplementary file 1 [file children-12-00308-s001.zip › children-3482635-supplementary.pdf]

Supplementary Table S1 Characteristics of study population

Variables	Boy (n =722)	Girl (n = 800)	Overall (n =1522)	*p* value
Age, y	12.3±3.5	12.7±3.5	12.5±3.5	0.022
BMI, kg/m2	19.8±3.7	19.4±3.33	19.6±3.5	0.025
Hight, cm	155.0±17.9	151.0±13.4	153.0±15.8	<0.001
BIA-VFA	28.5(18.9, 45.4)	46.5(29.2, 62.7)	37.2(22.9, 57.3)	<0.001
DXA-VFA	39.4 (33.4, 48.7)	27.5(19.4, 37.1)	34.6(25.3, 43.4)	<0.001

Abbreviations: BMI, body mass index; BIA-VFA, Visceral fat area was measured by bioelectric impedance analysis; DXA-VFA, Visceral fat area was measured by dual-energy x-ray absorptiometry.

Supplementary Table S2 Simplified thresholds of VFA for boys and girls aged 6 to 18 years

**Age (y)**	**DXA-VFA**	
	Boys	Girls
6~8	47.19	31.09
9~11	56.76	39.51
12~15	57.04	48.34
16~18	58.42	53.91

Abbreviations: DXA-VFA, visceral fat area was measured by dual-energy x-ray absorptiometry

Supplementary Table S3 The association of simplified and optimal cutoffs to identify CMR and its clustering after 2 years of follow-up in SCVBH

Type	Boy (RR95%CI)				Girl (RR95%CI)			
	Optimal cutoff	*p* value	Simplified cutoff	*p* value	Optimal cutoff	*p* value	Simplified cutoff	*p* value
**Hypertension**								
Total	2.91(2.56-3.32)	<0.001	3.04(2.67-3.46)	<0.001	2.99(2.58-3.46)	<0.001	2.93(2.53-3.39)	<0.001
Normal weight	2.32(0.86-5.79)	0.078	2.66(1.04-6.45)	<0.001	0.35(0.08-0.95)	0.077	0.67(0.26-1.45)	0.356
Overweight	1.24(0.99-1.56)	0.059	1.36(1.09-1.71)	0.007	1.52(1.15-2.03)	0.004	1.34(1.01-1.77)	0.041
Obesity	1.05(0.25-4.45)	0.950	2.93(0.99-10.65)	0.068	1.10(0.07-17.67)	0.948	1.10(0.07-17.67)	0.948
**Hyperglycemia**								
Total	1.33(1.11-1.58)	0.002	1.31(1.09-1.55)	0.002	1.64(1.30-2.05)	<0.001	1.63(1.30-2.05)	<0.001
Normal weight	2.09(0.59-5.80)	0.192	1.96(0.56-5.38)	0.232	1.00(0.05-5.17)	0.999	0.92(0.05-4.68)	0.934
Overweight	1.04(0.77-1.40)	0.817	0.98(0.73-1.33)	0.904	1.36(0.92-2.01)	0.121	1.32(0.89-1.95)	0.169
Obesity	1.19(0.21-22.39)	0.870	1.11(0.3-7.14)	0.895	1.07(0.13-9.04)	0.952	2.18(0.28-17.10)	0.459
**Dyslipidemia**								
Total	3.46(3.00-3.99)	<0.001	3.36(2.92-3.88)	<0.001	1.98(1.62-2.41)	<0.001	1.99(1.63-2.42)	<0.001
Normal weight	1.38(0.32-4.18)	0.608	1.85(0.53-5.08)	0.274	2.02(0.82-4.26)	0.089	1.80(0.74-3.77)	0.151
Overweight	1.68(1.32-2.15)	<0.001	1.50(1.18-1.92)	0.001	1.14(0.79-1.68)	0.481	1.18(0.82-1.72)	0.385
Obesity	4.34(0.43-35.45)	0.171	4.07(0.91-18.16)	0.066	NA		NA	
**CMR≥2**								
Total	3.82(3,23-4,51)	<0.001	3,89(3.30-4.60)	<0.001	3.23(2.54-4.10)	<0.001	3.16(2.48-4.01)	<0.001
Normal weight	3.66(1.04-10.20)	0.022	3.43(1.13-10.35)	0.029	0.52(0.03-2.39)	0.516	0.96(0.16-3.14)	0.958
Overweight	1.42(1.07-1.89)	0.015	1.44(1.08-1.91)	0.012	1.81(1.10-3.10)	0.025	1.53(0.95-2.53)	0.089
Obesity	NA		NA		NA		NA	

Abbreviations: CMR, cardiometabolic risk; RR, relative risk; SCVBH, The School-based Cardiovascular and Bone Health Promotion Program.

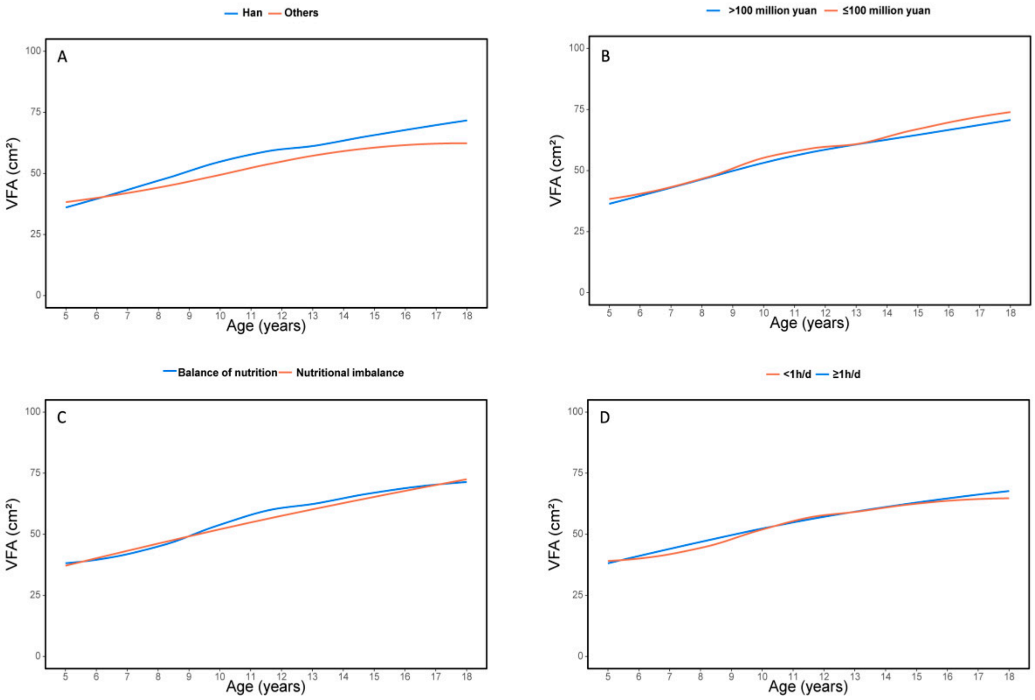

Supplementary Figure S1 The subgroup analysis of the DXA-based optimal VFA risk cutoffs

(A) The DXA-based optimal VFA risk cutoffs in different ethnic groups ($p<0.01$).

(B) The DXA-based optimal VFA risk cutoffs in different income ($p>0.05$).

(C) The DXA-based optimal VFA risk cutoffs in different diets ($p>0.05$).

(D) The DXA-based optimal VFA risk cutoffs in different physical activities ($p>0.05$).

Ethnic groups were classified into Han and minority populations (Han: 90.0%). Family income was categorized based on annual income: greater than 100,000 yuan and less than or equal to 100,000 yuan(>100 million yuan: 24%). Dietary classifications were based on the consumption of five food categories over the past month: 1. Vegetables or fruits ⩾ 1 time/day; 2. Meat and meat products (pork, beef, lamb, and poultry) ⩾ 2 times/week; 3. Dairy products ⩾ 1 time/day; 4. Soy products ⩾ 1 time/day; 5. Sugary beverages < 1 time/week. Individuals meeting four or more of these criteria were classified as meeting dietary standards/balance of nutrition; those who did not were classified as not meeting standards/nutritional imbalance (Dietary standard: 67.5%). Physical activity was defined as sufficient if individuals engaged in an average of 60 minutes or more of moderate to vigorous exercise per day over the past month; otherwise, it was considered insufficient(>1h/d: 23.6%).

Abbreviations: VFA, visceral fat area.

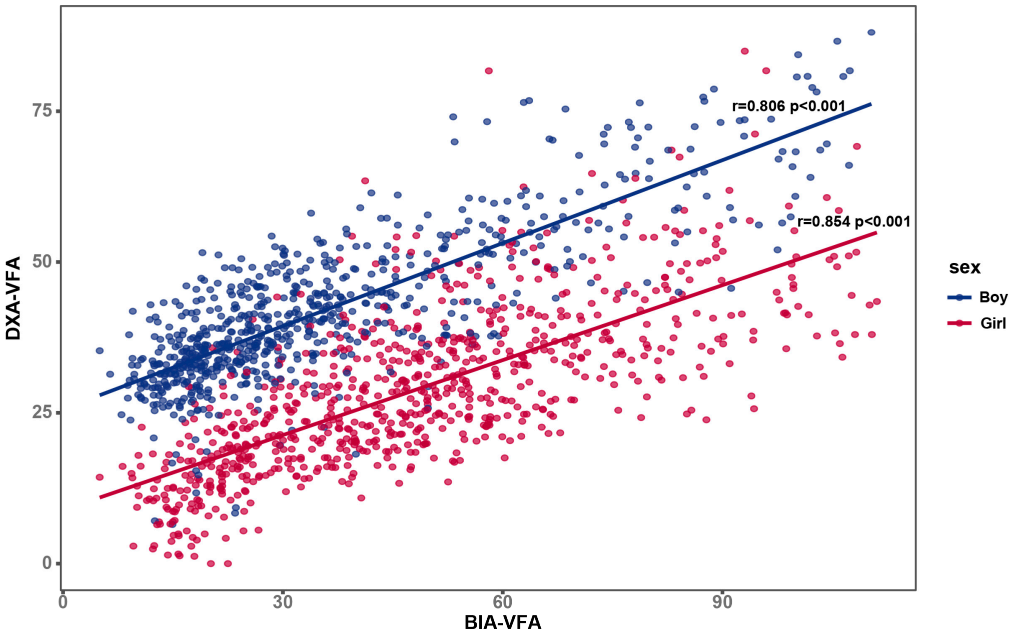

Supplementary Figure S2 Scatter plot of sex-specific VFA trends between DXA and BIA measurements

Abbreviations: BIA-VFA, Visceral fat area was measured by bioelectric impedance analysis; DXA-VFA, Visceral fat area was measured by dual-energy x-ray absorptiometry.

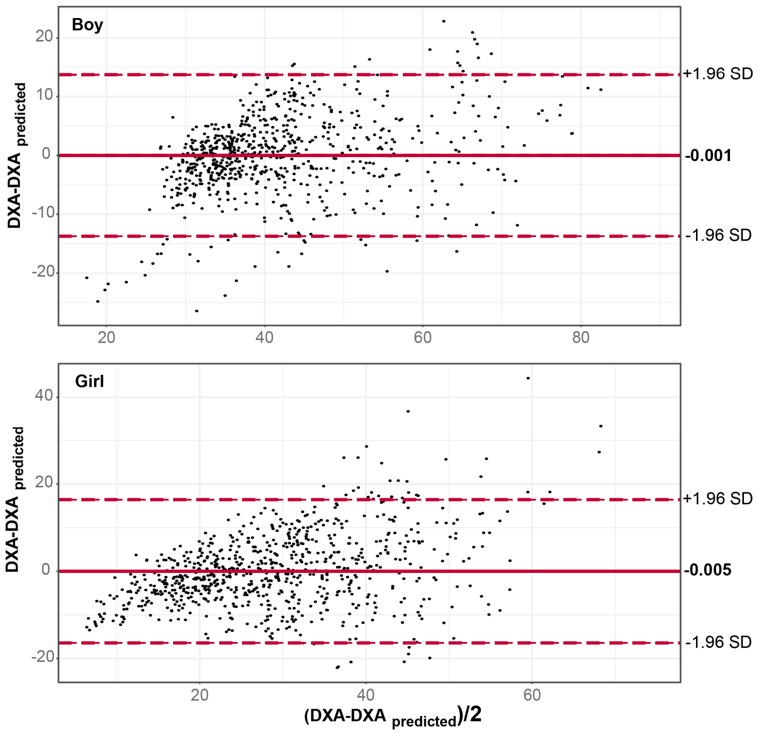

Supplementary Figure S3 Bland–Altman analysis comparing the difference between VFA from DXA and predicted DXA (n=1522)

Abbreviations: DXA, dual-energy x-ray absorptiometry.
